# Mapping the evolution of bornaviruses across geological timescales

**DOI:** 10.1073/pnas.2108123118

**Published:** 2021-06-04

**Authors:** Robert J. Gifford

**Affiliations:** ^a^Medical Research Council–University of Glasgow Centre for Virus Research, University of Glasgow, Glasgow G61 1QH, United Kingdom

It has always seemed likely that viruses originated early in the history of life. However, until the identification of endogenous viral elements (EVEs), there was little if any direct evidence for most virus groups ever having existed in the distant past (1). EVEs are virus-derived DNA sequences found in the germline genomes of metazoan species. Uniquely, they preserve information about the genomes of viruses that circulated tens to hundreds of millions of years ago. Comparative analysis of EVE sequences has now provided robust age calibrations for a diverse range of virus groups, completely transforming perspectives on the longer-term evolutionary interactions between viruses and hosts (2). As progress in mapping the complete genome sequences of species has accelerated, the abundance of “fossilized” viral sequences in eukaryotic genomes has become apparent. Increasingly, the challenge is not finding EVEs, but scaling analytical approaches to tackle the exponentially increasing volume of EVE sequence data (3, 4). In PNAS, Kawasaki et al. (5) utilize sophisticated computational approaches to implement a broad-scale analysis of the viral “fossil record,” focusing on a group of viruses called “bornaviruses.”

Bornaviruses (family *Bornaviridae*) are a poorly understood group of single-stranded negative sense RNA viruses (order *Mononegavirales*) that infect vertebrates. Until 2015 the family contained only one genus (*Orthobornavirus*); however, recent progress in sampling viral diversity has led to the establishment of two novel genera (*Carbovirus* and *Curtervirus*) (6). The prototype member, Borna disease virus (BDV), infects a variety of mammalian species and is the causative agent of a neurological condition in horses referred to as Borna disease or “sad horse disease.” The name “Borna” derives from an 1895 outbreak that occurred in the vicinity of the town of Borna in Saxony, Germany, and decimated the Prussian cavalry (7). The association of BDV with neurological disorders in mammals, combined with reports [now well established (8)] of zoonotic infections in humans, has driven a decades-long and frequently controversial effort to assess the role of bornavirus infection in human mental health conditions such as schizophrenia and depression (9).

A zoonotic transfer event involving a novel bornavirus was recently recorded in Germany following the deaths of three men from progressive encephalitis. Strikingly, all three were also breeders of exotic squirrels, and this epidemiological connection soon led investigators to identify the pathogen responsible—variegated squirrel bornavirus 1 (VSBV-1) (10). Retrospective studies have now established that VSBV-1 is an emerging pathogen that was introduced into the German captive squirrel population via exotic pet trade, probably around 2003, and has caused the deaths of at least four people (11).

**Fig. 1.**
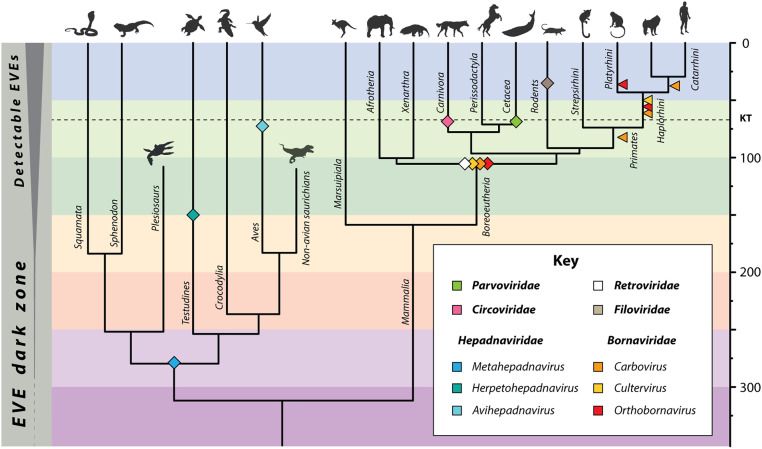
Timeline of vertebrate virus evolution based on direct evidence from the genomic fossil record. Minimum age estimates for various vertebrate virus lineages, as derived from identification of orthologous EVEs in related host taxa. Bornavirus-specific calibrations are taken from Kawasaki et al. (5) and are shown in relation to those obtained for circoviruses (1) (family *Circoviridae*), filoviruses (family *Filoviridae*) (1), hepadnaviruses (family *Hepadnaviridae*) (3), parvoviruses (family *Parvoviridae*) (19), and retroviruses (family *Retroviridae*) (20).

Information archived in EVE sequences can reveal the long-term evolutionary history of viruses, leading to a more complete understanding of their biology. The distribution and diversity of bornavirus EVEs have been investigated previously, but no previous study approaches the scale of that performed by Kawasaki et al. They searched the genome sequences of 969 eukaryotic species for bornaviral EVEs and identified hundreds of unique loci. Most of these bornavirus “fossils” are highly degraded and fragmentary, making it difficult to do meaningful comparative analyses. However, by artfully combining high-throughput computational approaches with careful human oversight, Kawasaki et al. tease out the evolutionary connections between contemporary bornaviruses and the extinct “paleoviruses” represented by EVE sequences, thereby reconstructing a detailed picture of bornavirus evolution (Fig. 1). Numerous novel subgroups were discovered, some of which may represent novel genera. In addition, EVEs show that the host range of extant bornavirus genera is much broader than expected, encompassing multiple vertebrate classes.

Of all the kinds of inferences that can be extracted from EVE sequence data, age calibrations based on the detection of orthologous EVEs in related species are among the most reliable, since they are derived in part from an orthogonal data source (the fossil record of host species) (2). However, identifying EVE insertions that share homologous genomic flanks can be a labor-intensive process, particularly when using incomplete and fragmentary genome sequences. A further complicating factor is the tendency of EVEs to occur in repetitive genomic regions (12). In the PNAS study, an innovative, network-based approach was used to identify orthologous EVEs, enabling the authors to derive age estimates for large numbers of loci and broadly calibrate the timeline of bornavirus evolution. This revealed that stable relationships with bornaviruses have persisted over the long term in some host groups (notably primates), and that different viral lineages have apparently been prevalent during different time periods. In addition, correlating the timelines of host and viruses indicated that periods of biogeographic isolation experienced by mammalian groups during their evolution have played an important role in shaping bornavirus host range.

For an EVE sequence to arise, DNA derived from a virus genome has to become integrated into the genome of an infected germline cell. Current evidence indicates that in the case of bornaviruses and other mononegaviruses, integration usually occurs via LINE1-mediated retrotransposition: Viral messenger RNA (mRNA) becomes associated with a LINE1 protein complex, which leads to it being reverse-transcribed into DNA and integrated into the nuclear genome (12). A telltale sign of this mechanism of integration is the presence of an adenine-rich poly-A tail and target site duplication sequences flanking the integrated EVE (2, 4). In the case of mononegaviruses, there seems to be a strong bias toward integration of mRNAs derived from the nucleoprotein (N) gene, which is only one of six proteins encoded by bornavirus genomes. Fossilized copies of four other genes were also identified, but at considerably lower frequency. The observation that the divergent accessory (X) protein is not detected, and that less conserved genes are generally detected at lower levels, raises the possibility that some bornavirus-derived EVEs might be unrecognizable as such due to high levels of sequence divergence [although they may still be recognizable as EVEs (4)], especially those that are older and derived from more rapidly evolving genes.

The frequency with which bornavirus EVEs occur in certain vertebrate groups raises questions about their potential functional roles as co-opted or exapted host genes. Previous work has found evidence that some have antiviral functions, and conceivably bornaviral EVEs could be involved in other biological processes (12–15). Practically all EVEs that have so far been described are genetically “fixed” (i.e., they occur at 100% frequency in the host gene pool), and while almost all are degraded by mutation, some show signs of potentially having been co-opted in the past (1, 13). Conceivably, situations may occur in which integrated EVEs confer a selective benefit to the host, but the timeframe of functional relevance is significantly shorter than the time required to reach fixation. Under these circumstances, important functional roles for EVEs might be occulted to our current methods of investigation.

The PNAS study illustrates some of the limitations of nonretroviral EVE data. Even though bornavirus-derived EVEs are quite common in some groups, they are sparsely and patchily distributed overall. This can make it quite difficult to interpret the relationship between EVE distribution and diversity and long-term virus–host interactions. For example, the absence of EVEs from some species groups might superficially seem to imply the historical absence of virus, but other factors impacting the distribution of EVEs need to be considered. These are not restricted to the biological aspects of virus replication that increase the likelihood of germline integration (e.g., access to germline cells, replication within the nucleus)—all of which could potentially vary greatly among virus strains and host species—but also encompass a broad range of factors influencing the frequency with which integrated EVE sequences become fixed in the germline. Straightforward population genetics laws dictate that the vast majority of EVEs will be eliminated from the gene pool within a few host generations. Consequently, factors impacting the likelihood of fixation (e.g., host population size) could potentially have played an important role in shaping EVE distributions, and this may distort our impressions.

While EVE-based studies have limitations, there is no denying their fundamental impact on our understanding of virus evolution. Increasingly, this is being reflected in studies of contemporary viruses as date calibrations allow species diversity and biological properties to be placed into evolutionary context (6, 16). In addition, mapping the genomic fossil record of viruses can help enable “paleovirology”—i.e., empirical studies of paleoviruses. Recent studies have shown that EVE sequences can be used to guide the reconstitution of functional versions of paleovirus proteins, thereby allowing their biological properties to be explored in vitro (17, 18). The detailed mapping of bornavirus EVEs performed by Kawasaki et al. can provide a basis for future work in this direction.
